# Supplementary material for: Reconciling Mining with the Conservation of Cave Biodiversity: A Quantitative Baseline to Help Establish Conservation Priorities
Source: PLoS One. 2016 Dec 20;11(12):e0168348. doi: 10.1371/journal.pone.0168348 (PMC5173368; doi:10.1371/journal.pone.0168348)
Supplement: S1 Dataset — (ZIP) [file pone.0168348.s002.zip › Taxa/Serra Norte/SN_2013/N5S/Lista N5S-49.pdf]

CAVIDADE N5S-0049

Classe	Ordem	Fam/Outros	Gên/Outros	Espécie	S-U	U-S	Zon.
Annelida	Oligochaeta			jovem	1		E
Arachnida	Araneae	Pholcidae		jovem		X	E
Arachnida	Araneae	Pisauridae		jovem	1	1	E
Arachnida	Araneae	Theridiosomatidae	*Plato*	sp.1		X	E
Arachnida	Opiliones	Sclerosomatidae	*Prionostema*	sp.1	3		E
Entognatha	Collembola	Entomobryidae		sp.1	X		E
Entognatha	Collembola	Entomobryidae		sp.6	X		E
Entognatha	Collembola	Paronellidae		sp.1	X		E
Insecta	Coleoptera	Dytiscidae		sp.5	X	X	E
Insecta	Coleoptera	Hydrophilidae		sp.3	X		E
Insecta	Coleoptera	Staphylinidae	Pselaphinae	sp.10	X		E
Insecta	Diptera	Culicidae	*Culex*	sp.	X		E
Insecta	Diptera	Keroplatidae	*Euceroplatus*	sp.	X		E
Insecta	Diptera	Tipulidae	Tipulinae	sp.	X	X	E
Insecta	Heteroptera	Belostomatidae		sp.	X	X	E
Insecta	Heteroptera	Veliidae	*Paravelia*	sp.		X	E
Insecta	Heteroptera	Veliidae	*Rhagovelia*	sp.	X		E
Insecta	Hymenoptera	Formicidae	*Paratrechina*	sp.1	X		E
Insecta	Hymenoptera	Formicidae	*Solenopsis*	sp.2	X		E
Insecta	Isoptera	Termitidae	*Nasutitermes*	sp.	X		E
Insecta	Orthoptera	Phalangopsidae	*Paraclodes*	sp.		3	E
Insecta	Psocoptera	Epipsocidae	*Epipsocus*	sp.2	X		E
Nematoda				sp.1	X		E
Amphibia	Anura	Strabomantidae	*Pristimantis*	cf. *fenestratus*	1		E
